# Bis[tris(3-*tert*-butyl-5-methyl­pyrazol-1-yl)hydridoborato]ytterbium(II) toluene solvate

**DOI:** 10.1107/S1600536809017152

**Published:** 2009-05-14

**Authors:** Kuburat O. Saliu, Josef Takats, Michael J. Ferguson

**Affiliations:** a Department of Chemistry, University of Alberta, Edmonton, Alberta, Canada T6G 2G2; b X-ray Crystallography Laboratory, Department of Chemistry, University of Alberta, Edmonton, Alberta, Canada T6G 2G2

## Abstract

In the title compound, [Yb(C_24_H_40_BN_6_)_2_]·C_7_H_8_, the Yb atom is coordinated by two tris(3-*tert*-butyl-5-methyl­pyrazol-1-yl)hydridoborate [Tp^*t*Bu,Me^] ligands. One ligand binds in the κ^3^ mode, throuh three N atoms of the pyrazolyl rings, the other ligand coordinates through two N atoms of the pyrazolyl rings and the H atom attached to the central B *via* an agostic-type inter­action through the B—H group of the second Tp^*t*Bu,Me^ ligand, giving an overall distorted octa­hedral geometry. One of the *tert*-butyl groups is disordered over two sites, with occupancies of 0.65 and 0.35.

## Related literature

For full details of the synthesis and spectroscopic characterization of the title compound, see: Zhang *et al.* (1995 ▶). For the samarium analogue, see: Zhang *et al.* (1995 ▶). For the thulium analogue, see: Cheng *et al.* (2008 ▶). For B—H agostic inter­actions involving pyrazolylborate ligands, see: Calabrese *et al.* (1990 ▶); Kosky *et al.* (1971 ▶); Cotton *et al.* (1972 ▶).
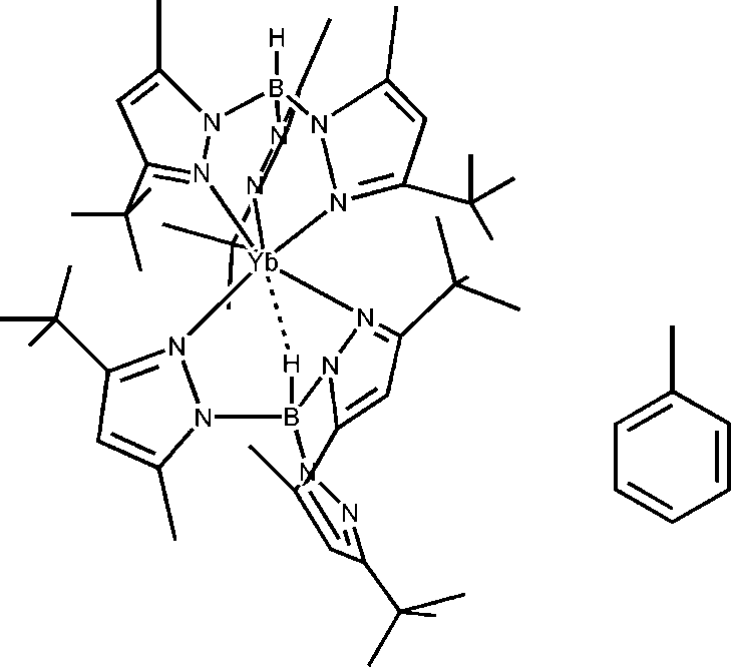


## Experimental

### 

#### Crystal data

[Yb(C_24_H_40_BN_6_)_2_]·C_7_H_8_
*M* *_r_* = 1112.03
Triclinic, 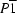

*a* = 11.8621 (17) Å
*b* = 12.1587 (17) Å
*c* = 20.650 (3) Å
α = 96.1072 (19)°
β = 96.1001 (18)°
γ = 98.9737 (19)°
*V* = 2902.1 (7) Å^3^
*Z* = 2
Mo *K*α radiation
μ = 1.66 mm^−1^
*T* = 193 K
0.39 × 0.36 × 0.14 mm

#### Data collection

Bruker SMART 1000 diffractometer
Absorption correction: integration (*SHELXTL*; Sheldrick, 2008 ▶) *T*_min_ = 0.564, *T*_max_ = 0.801
24467 measured reflections
13091 independent reflections
11816 reflections with *I* > 2σ(*I*)
*R*_int_ = 0.026

#### Refinement

*R*[*F* ^2^ > 2σ(*F* ^2^)] = 0.035
*wR*(*F* ^2^) = 0.096
*S* = 1.10
13091 reflections
669 parameters
H atoms treated by a mixture of independent and constrained refinement
Δρ_max_ = 1.91 e Å^−3^
Δρ_min_ = −1.74 e Å^−3^

### 

Data collection: *SMART* (Bruker, 1997 ▶); cell refinement: *SAINT* (Bruker, 1997 ▶); data reduction: *SAINT*; program(s) used to solve structure: *SHELXS97* (Sheldrick, 2008 ▶); program(s) used to refine structure: *SHELXL97* (Sheldrick, 2008 ▶); molecular graphics: *SHELXTL* (Sheldrick, 2008 ▶); software used to prepare material for publication: *SHELXTL*.

## Supplementary Material

Crystal structure: contains datablocks I, global. DOI: 10.1107/S1600536809017152/lh2812sup1.cif

Structure factors: contains datablocks I. DOI: 10.1107/S1600536809017152/lh2812Isup2.hkl

Additional supplementary materials:  crystallographic information; 3D view; checkCIF report

## Figures and Tables

**Table 1:** Selected geometric parameters (Å, °)

Yb—N32	2.477 (3)
Yb—N12	2.524 (3)
Yb—N22	2.547 (2)
Yb—N42	2.555 (2)
Yb—N52	2.632 (3)
Yb—N41	2.990 (2)
Yb—H2*B*	2.29 (4)
N11—B1	1.554 (4)
N21—B1	1.558 (4)
N31—B1	1.560 (4)
N41—B2	1.546 (4)
N51—B2	1.553 (4)
N61—B2	1.527 (4)
B2—H2*B*	1.16 (3)

**Table 2:** Hydrogen-bond geometry (Å, °)

*D*—H⋯*A*	*D*—H	H⋯*A*	*D*⋯*A*	*D*—H⋯*A*
B2—H2*B*⋯Yb	1.16 (3)	2.29 (4)	3.002 (3)	118 (2)

**Fig. 1.**
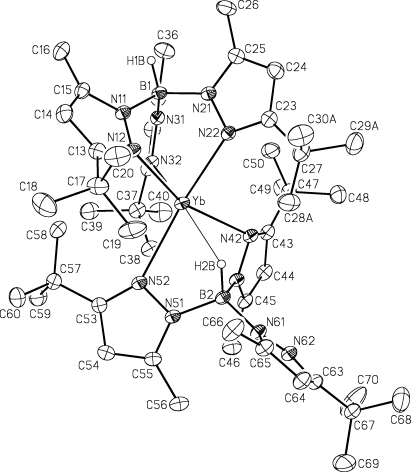
The molecular structure of the title compound, showing the atom labelling scheme. Non-hydrogen atoms are represented by ellipsoids at the 30% probability level. H atoms attached to B atoms are shown with arbitrarily small radii; the remaining H atoms are not shown for clarity. The disorder and the solvent molecule are not shown.

## supplementary crystallographic information

### Comment

The synthesis, solution structure and spectroscopic characterization of the title compound was reported by Zhang *et al.* (1995) along with the samarium analogue whose structure was reported then. The structure of the ytterbium analogue is similar to that reported for the samarium compound featuring a distorted six coordinate octahedral geometry formed by five N atoms of two Tp*^t^*^Bu,Me^ ligands and additional agostic B—H interaction from the B—H group of one Tp*^t^*^Bu,Me^ ligand. The axial sites are occupied by N32 and H2B. The distortion from ideal geometry is obvious from the deviation of the interatomic angles and distances from the idealized values. The intraligand equatorial bonds to the axial N32 are approximately 90° (87.15 (8)° and 93.28 (8)° to N12 and N22, respectively) whereas the corresponding angles to the inter-ligand equatorial N atoms are greater than 90° (97.77 (8)° and 108.92 (8)°, respectively). The distortion from the ideal geometry is also reflected in the non-lineal axial angle of 153.8 (9)°, which is comparable to the value of 152° observed in the samarium analogue. The presence of the B—H agostic interaction is evident in the short Yb—B2 interatomic distance of 3.002 (3) Å, compared to the much longer Yb—B1 separation of 3.310 (3) Å in the unperturbed ligand. Further, the average Yb—N bond distance in the κ^3^ ligand is shorter than that in the formally κ^2^ ligand, 2.52 (2) Å and 2.59 (4) Å, respectively.

### Experimental

X-ray quality crystals of the titled compound were obtained *via* ligand redistribution while attempting to grow crystals of (Tp*^t^*^Bu,Me^)Yb(BH_4_) from a dilute toluene solution cooled to 233 K over several days.

### Refinement

H atom H2B (attached to B atom B2) was allowed to freely refine; the remaining H atoms were generated in idealized positions (according to the *sp*^2^ or *sp*^3^ geometries of their parent C or B atoms), and then refined using a riding model with fixed C—H and B—H distances (C—H = 0.95–1.00 Å, B1—H1B = 1.00 Å) and with *U*_iso_(H) = 1.2*U*_eq_(C,B). One of the t-butyl groups is disordered over two sites with initially refined occupancies which were finally fixed at 0.65 and 0.35.

### Crystal data

**Table:**

[Yb(C_24_H_40_BN_6_)_2_]·C_7_H_8_	*Z* = 2
*M**_r_* = 1112.03	*F*(000) = 1164
Triclinic, *P*1	*D*_x_ = 1.273 Mg m^−^^3^
Hall symbol: -P 1	Mo *K*α radiation, λ = 0.71073 Å
*a* = 11.8621 (17) Å	Cell parameters from 6598 reflections
*b* = 12.1587 (17) Å	θ = 2.2–27.5°
*c* = 20.650 (3) Å	µ = 1.66 mm^−^^1^
α = 96.1072 (19)°	*T* = 193 K
β = 96.1001 (18)°	Block, yellow
γ = 98.9737 (19)°	0.39 × 0.36 × 0.14 mm
*V* = 2902.1 (7) Å^3^	

### Data collection

**Table:**

Bruker PLATFORM diffractometer with SMART 1000 CCD area-detector	13091 independent reflections
Radiation source: fine-focus sealed tube	11816 reflections with *I* > 2σ(*I*)
graphite	*R*_int_ = 0.026
Detector resolution: 8.192 pixels mm^-1^	θ_max_ = 27.6°, θ_min_ = 1.7°
ω scans	*h* = −15→15
Absorption correction: integration (*SHELXTL*; Sheldrick, 2008)	*k* = −15→15
*T*_min_ = 0.564, *T*_max_ = 0.801	*l* = −26→26
24467 measured reflections	

### Refinement

**Table:**

Refinement on *F*^2^	Primary atom site location: structure-invariant direct methods
Least-squares matrix: full	Secondary atom site location: difference Fourier map
*R*[*F*^2^ > 2σ(*F*^2^)] = 0.035	Hydrogen site location: inferred from neighbouring sites
*wR*(*F*^2^) = 0.096	H atoms treated by a mixture of independent and constrained refinement
*S* = 1.10	*w* = 1/[σ^2^(*F*_o_^2^) + (0.0559*P*)^2^ + 1.1666*P*] where *P* = (*F*_o_^2^ + 2*F*_c_^2^)/3
13091 reflections	(Δ/σ)_max_ = 0.001
669 parameters	Δρ_max_ = 1.91 e Å^−^^3^
0 restraints	Δρ_min_ = −1.74 e Å^−^^3^

### Special details

**Table:**

Geometry. All e.s.d.'s (except the e.s.d. in the dihedral angle between two l.s. planes) are estimated using the full covariance matrix. The cell e.s.d.'s are taken into account individually in the estimation of e.s.d.'s in distances, angles and torsion angles; correlations between e.s.d.'s in cell parameters are only used when they are defined by crystal symmetry. An approximate (isotropic) treatment of cell e.s.d.'s is used for estimating e.s.d.'s involving l.s. planes.
Refinement. Refinement of *F*^2^ against ALL reflections. The weighted *R*-factor *wR* and goodness of fit *S* are based on *F*^2^, conventional *R*-factors *R* are based on *F*, with *F* set to zero for negative *F*^2^. The threshold expression of *F*^2^ > σ(*F*^2^) is used only for calculating *R*-factors(gt) *etc*. and is not relevant to the choice of reflections for refinement. *R*-factors based on *F*^2^ are statistically about twice as large as those based on *F*, and *R*- factors based on ALL data will be even larger.

### Fractional atomic coordinates and isotropic or equivalent isotropic displacement parameters (Å^2^)

**Table:**

	*x*	*y*	*z*	*U*_iso_*/*U*_eq_	Occ. (<1)
Yb	0.234066 (10)	0.515202 (10)	0.265241 (5)	0.02764 (5)	
N11	0.3907 (2)	0.6798 (2)	0.38158 (12)	0.0297 (5)	
N12	0.2745 (2)	0.6378 (2)	0.37444 (13)	0.0336 (5)	
N21	0.4464 (2)	0.4848 (2)	0.37392 (12)	0.0298 (5)	
N22	0.3382 (2)	0.4220 (2)	0.35293 (12)	0.0284 (5)	
N31	0.5013 (2)	0.6172 (2)	0.28709 (12)	0.0292 (5)	
N32	0.4151 (2)	0.6195 (2)	0.23690 (12)	0.0298 (5)	
N41	0.1149 (2)	0.3613 (2)	0.14689 (12)	0.0285 (5)	
N42	0.2164 (2)	0.3423 (2)	0.18056 (12)	0.0295 (5)	
N51	−0.0193 (2)	0.4987 (2)	0.17664 (12)	0.0300 (5)	
N52	0.0614 (2)	0.5913 (2)	0.20427 (12)	0.0301 (5)	
N61	−0.0833 (2)	0.2865 (2)	0.17723 (12)	0.0309 (5)	
N62	−0.0989 (2)	0.2068 (2)	0.12360 (12)	0.0319 (5)	
C13	0.2270 (3)	0.7081 (3)	0.41270 (15)	0.0358 (7)	
C14	0.3108 (3)	0.7970 (3)	0.44379 (16)	0.0401 (7)	
H14	0.2993	0.8586	0.4732	0.048*	
C15	0.4133 (3)	0.7774 (3)	0.42319 (14)	0.0333 (6)	
C16	0.5295 (3)	0.8479 (3)	0.43985 (18)	0.0443 (8)	
H16A	0.5523	0.8812	0.4010	0.053*	
H16B	0.5853	0.8012	0.4542	0.053*	
H16C	0.5273	0.9078	0.4752	0.053*	
C17	0.1020 (3)	0.6831 (3)	0.42488 (16)	0.0439 (8)	
C18	0.0510 (4)	0.7912 (5)	0.4291 (2)	0.0684 (13)	
H18A	−0.0298	0.7743	0.4365	0.082*	
H18B	0.0561	0.8241	0.3880	0.082*	
H18C	0.0940	0.8446	0.4656	0.082*	
C19	0.0306 (3)	0.5959 (5)	0.3714 (2)	0.0634 (13)	
H19A	−0.0494	0.5822	0.3807	0.076*	
H19B	0.0617	0.5258	0.3706	0.076*	
H19C	0.0336	0.6239	0.3287	0.076*	
C20	0.0994 (3)	0.6359 (4)	0.49101 (19)	0.0540 (10)	
H20A	0.0197	0.6198	0.5005	0.065*	
H20B	0.1447	0.6912	0.5259	0.065*	
H20C	0.1322	0.5666	0.4888	0.065*	
C23	0.3343 (3)	0.3281 (3)	0.38186 (15)	0.0342 (6)	
C24	0.4389 (3)	0.3287 (3)	0.42021 (17)	0.0406 (7)	
H24	0.4587	0.2717	0.4452	0.049*	
C25	0.5073 (3)	0.4286 (3)	0.41436 (16)	0.0365 (7)	
C26	0.6280 (3)	0.4703 (3)	0.44679 (19)	0.0484 (9)	
H26A	0.6471	0.4230	0.4805	0.058*	
H26B	0.6343	0.5479	0.4671	0.058*	
H26C	0.6815	0.4672	0.4139	0.058*	
C27	0.2263 (3)	0.2412 (3)	0.37726 (17)	0.0412 (7)	
C28A	0.1367 (6)	0.2548 (6)	0.3271 (3)	0.0577 (17)	0.65
H28A	0.1684	0.2600	0.2854	0.069*	0.65
H28B	0.1066	0.3234	0.3398	0.069*	0.65
H28C	0.0745	0.1900	0.3221	0.069*	0.65
C29A	0.2611 (6)	0.1207 (5)	0.3635 (4)	0.0610 (17)	0.65
H29A	0.3170	0.1097	0.3997	0.073*	0.65
H29B	0.2953	0.1152	0.3224	0.073*	0.65
H29C	0.1924	0.0630	0.3600	0.073*	0.65
C30A	0.1861 (6)	0.2466 (7)	0.4457 (3)	0.0609 (17)	0.65
H30A	0.2526	0.2525	0.4791	0.073*	0.65
H30B	0.1315	0.1784	0.4483	0.073*	0.65
H30C	0.1486	0.3123	0.4532	0.073*	0.65
C28B	0.1896 (12)	0.1808 (11)	0.3063 (6)	0.060 (3)	0.35
H28D	0.1921	0.2365	0.2753	0.072*	0.35
H28E	0.1111	0.1389	0.3029	0.072*	0.35
H28F	0.2423	0.1288	0.2959	0.072*	0.35
C29B	0.2290 (12)	0.1627 (12)	0.4256 (7)	0.067 (4)	0.35
H29D	0.1562	0.1098	0.4192	0.080*	0.35
H29E	0.2400	0.2040	0.4698	0.080*	0.35
H29F	0.2927	0.1212	0.4203	0.080*	0.35
C30B	0.1189 (10)	0.3114 (11)	0.3882 (7)	0.057 (3)	0.35
H30D	0.1209	0.3704	0.3594	0.069*	0.35
H30E	0.1271	0.3453	0.4341	0.069*	0.35
H30F	0.0455	0.2597	0.3778	0.069*	0.35
C33	0.4685 (3)	0.6383 (3)	0.18325 (15)	0.0335 (6)	
C34	0.5866 (3)	0.6460 (3)	0.19885 (16)	0.0397 (7)	
H34	0.6434	0.6581	0.1700	0.048*	
C35	0.6052 (3)	0.6327 (3)	0.26406 (16)	0.0354 (7)	
C36	0.7174 (3)	0.6337 (4)	0.3053 (2)	0.0513 (9)	
H36A	0.7164	0.5616	0.3226	0.062*	
H36B	0.7287	0.6943	0.3419	0.062*	
H36C	0.7804	0.6458	0.2784	0.062*	
C37	0.4057 (3)	0.6577 (3)	0.11836 (16)	0.0392 (7)	
C38	0.2797 (3)	0.6037 (3)	0.10966 (16)	0.0425 (8)	
H38A	0.2425	0.6172	0.0672	0.051*	
H38B	0.2416	0.6363	0.1451	0.051*	
H38C	0.2738	0.5227	0.1111	0.051*	
C39	0.4161 (4)	0.7848 (4)	0.1163 (2)	0.0567 (10)	
H39A	0.3771	0.7988	0.0745	0.068*	
H39B	0.4975	0.8187	0.1206	0.068*	
H39C	0.3803	0.8181	0.1526	0.068*	
C40	0.4633 (4)	0.6068 (5)	0.06140 (19)	0.0675 (13)	
H40A	0.4234	0.6192	0.0195	0.081*	
H40B	0.4592	0.5260	0.0631	0.081*	
H40C	0.5441	0.6428	0.0655	0.081*	
C43	0.2782 (3)	0.3039 (3)	0.13496 (15)	0.0323 (6)	
C44	0.2174 (3)	0.2991 (3)	0.07203 (15)	0.0361 (7)	
H44	0.2417	0.2749	0.0313	0.043*	
C45	0.1162 (3)	0.3364 (3)	0.08108 (14)	0.0330 (6)	
C46	0.0227 (3)	0.3551 (3)	0.03069 (16)	0.0435 (8)	
H46A	−0.0443	0.2959	0.0289	0.052*	
H46B	0.0504	0.3532	−0.0124	0.052*	
H46C	0.0008	0.4283	0.0426	0.052*	
C47	0.3911 (3)	0.2634 (3)	0.15263 (16)	0.0367 (7)	
C48	0.3633 (4)	0.1369 (3)	0.1577 (2)	0.0541 (9)	
H48A	0.4348	0.1085	0.1692	0.065*	
H48B	0.3246	0.0973	0.1155	0.065*	
H48C	0.3128	0.1243	0.1918	0.065*	
C49	0.4683 (4)	0.2803 (4)	0.0987 (2)	0.0608 (11)	
H49A	0.5406	0.2536	0.1105	0.073*	
H49B	0.4846	0.3603	0.0936	0.073*	
H49C	0.4292	0.2379	0.0572	0.073*	
C50	0.4547 (3)	0.3237 (4)	0.2175 (2)	0.0537 (10)	
H50A	0.5267	0.2952	0.2271	0.064*	
H50B	0.4066	0.3103	0.2526	0.064*	
H50C	0.4717	0.4044	0.2147	0.064*	
C53	0.0184 (3)	0.6813 (3)	0.18775 (14)	0.0317 (6)	
C54	−0.0897 (3)	0.6475 (3)	0.15046 (16)	0.0367 (7)	
H54	−0.1386	0.6949	0.1330	0.044*	
C55	−0.1110 (3)	0.5320 (3)	0.14419 (15)	0.0342 (6)	
C56	−0.2151 (3)	0.4535 (3)	0.1098 (2)	0.0494 (9)	
H56A	−0.2652	0.4285	0.1421	0.059*	
H56B	−0.1913	0.3883	0.0863	0.059*	
H56C	−0.2571	0.4923	0.0784	0.059*	
C57	0.0799 (3)	0.8017 (3)	0.20749 (16)	0.0386 (7)	
C58	0.1913 (3)	0.8086 (3)	0.25287 (19)	0.0451 (8)	
H58A	0.2257	0.8875	0.2662	0.054*	
H58B	0.2450	0.7703	0.2298	0.054*	
H58C	0.1749	0.7724	0.2918	0.054*	
C59	0.1047 (4)	0.8533 (3)	0.1446 (2)	0.0529 (9)	
H59A	0.1428	0.9315	0.1562	0.063*	
H59B	0.0322	0.8506	0.1164	0.063*	
H59C	0.1549	0.8107	0.1212	0.063*	
C60	−0.0021 (4)	0.8676 (3)	0.2430 (2)	0.0559 (10)	
H60A	0.0367	0.9449	0.2576	0.067*	
H60B	−0.0236	0.8314	0.2812	0.067*	
H60C	−0.0715	0.8683	0.2128	0.067*	
C63	−0.1930 (3)	0.1361 (3)	0.12911 (15)	0.0326 (6)	
C64	−0.2405 (3)	0.1687 (3)	0.18675 (16)	0.0383 (7)	
H64	−0.3075	0.1319	0.2019	0.046*	
C65	−0.1688 (3)	0.2649 (3)	0.21601 (15)	0.0361 (7)	
C66	−0.1746 (3)	0.3395 (3)	0.27764 (19)	0.0511 (9)	
H66A	−0.1673	0.4176	0.2685	0.061*	
H66B	−0.1118	0.3324	0.3110	0.061*	
H66C	−0.2486	0.3175	0.2938	0.061*	
C67	−0.2372 (3)	0.0362 (3)	0.07715 (16)	0.0371 (7)	
C68	−0.2400 (5)	−0.0718 (4)	0.1088 (3)	0.0722 (14)	
H68A	−0.2658	−0.1365	0.0751	0.087*	
H68B	−0.2933	−0.0728	0.1420	0.087*	
H68C	−0.1627	−0.0754	0.1297	0.087*	
C69	−0.3593 (5)	0.0404 (5)	0.0492 (3)	0.0818 (17)	
H69A	−0.3879	−0.0252	0.0164	0.098*	
H69B	−0.3610	0.1090	0.0284	0.098*	
H69C	−0.4082	0.0401	0.0845	0.098*	
C70	−0.1604 (5)	0.0318 (5)	0.0236 (3)	0.095 (2)	
H70A	−0.1889	−0.0355	−0.0079	0.114*	
H70B	−0.0819	0.0292	0.0428	0.114*	
H70C	−0.1606	0.0987	0.0010	0.114*	
B1	0.4809 (3)	0.6082 (3)	0.35989 (16)	0.0293 (6)	
H1B	0.5555	0.6397	0.3878	0.035*	
B2	0.0173 (3)	0.3843 (3)	0.18781 (16)	0.0279 (6)	
H2B	0.062 (3)	0.396 (3)	0.2413 (17)	0.032 (9)*	
C1S	0.7433 (4)	0.0308 (4)	0.3474 (2)	0.0631 (11)	
C2S	0.6933 (5)	−0.0606 (4)	0.3026 (2)	0.0618 (11)	
H2S	0.7379	−0.1156	0.2895	0.074*	
C3S	0.5813 (5)	−0.0732 (4)	0.2769 (2)	0.0708 (13)	
H3S	0.5484	−0.1382	0.2472	0.085*	
C4S	0.5155 (5)	0.0042 (5)	0.2924 (3)	0.0787 (15)	
H4S	0.4373	−0.0059	0.2737	0.094*	
C5S	0.5635 (6)	0.0986 (5)	0.3358 (3)	0.0779 (15)	
H5S	0.5190	0.1550	0.3462	0.093*	
C6S	0.6761 (5)	0.1104 (4)	0.3640 (2)	0.0647 (12)	
H6S	0.7080	0.1739	0.3951	0.078*	
C7S	0.8648 (6)	0.0440 (7)	0.3760 (4)	0.127 (3)	
H7SA	0.9102	0.0164	0.3427	0.152*	
H7SB	0.8706	0.0008	0.4134	0.152*	
H7SC	0.8945	0.1234	0.3910	0.152*	

### Atomic displacement parameters (Å^2^)

**Table:**

	*U*^11^	*U*^22^	*U*^33^	*U*^12^	*U*^13^	*U*^23^
Yb	0.02238 (7)	0.03397 (8)	0.02434 (7)	0.00150 (5)	0.00309 (4)	−0.00177 (5)
N11	0.0299 (13)	0.0335 (13)	0.0243 (11)	0.0036 (10)	0.0042 (9)	−0.0006 (9)
N12	0.0279 (13)	0.0408 (14)	0.0299 (12)	0.0057 (11)	0.0026 (10)	−0.0037 (10)
N21	0.0264 (12)	0.0340 (13)	0.0278 (11)	0.0037 (10)	0.0033 (9)	0.0014 (9)
N22	0.0244 (12)	0.0296 (12)	0.0301 (12)	0.0008 (10)	0.0056 (9)	0.0018 (9)
N31	0.0242 (12)	0.0309 (12)	0.0322 (12)	0.0033 (10)	0.0074 (9)	0.0006 (10)
N32	0.0285 (13)	0.0329 (12)	0.0267 (11)	0.0003 (10)	0.0068 (9)	0.0017 (9)
N41	0.0249 (12)	0.0331 (13)	0.0263 (11)	0.0007 (10)	0.0052 (9)	0.0020 (9)
N42	0.0250 (12)	0.0337 (13)	0.0279 (12)	0.0019 (10)	0.0049 (9)	−0.0020 (9)
N51	0.0245 (12)	0.0345 (13)	0.0289 (12)	0.0012 (10)	0.0028 (9)	0.0006 (10)
N52	0.0253 (12)	0.0344 (13)	0.0283 (12)	0.0006 (10)	0.0035 (9)	0.0001 (10)
N61	0.0268 (12)	0.0333 (13)	0.0305 (12)	−0.0014 (10)	0.0082 (10)	−0.0002 (10)
N62	0.0287 (13)	0.0346 (13)	0.0301 (12)	0.0002 (10)	0.0052 (10)	−0.0004 (10)
C13	0.0384 (17)	0.0443 (17)	0.0261 (14)	0.0124 (14)	0.0073 (12)	−0.0013 (12)
C14	0.049 (2)	0.0404 (17)	0.0315 (15)	0.0105 (15)	0.0098 (14)	−0.0035 (13)
C15	0.0418 (17)	0.0312 (15)	0.0255 (13)	0.0026 (13)	0.0060 (12)	0.0008 (11)
C16	0.050 (2)	0.0354 (17)	0.0430 (18)	−0.0054 (15)	0.0078 (15)	0.0014 (14)
C17	0.0340 (17)	0.065 (2)	0.0340 (16)	0.0173 (16)	0.0083 (13)	−0.0032 (15)
C18	0.066 (3)	0.091 (4)	0.061 (3)	0.044 (3)	0.021 (2)	0.007 (2)
C19	0.0305 (19)	0.103 (4)	0.049 (2)	0.006 (2)	0.0065 (16)	−0.018 (2)
C20	0.039 (2)	0.079 (3)	0.0436 (19)	0.0042 (19)	0.0140 (15)	0.0048 (18)
C23	0.0369 (17)	0.0362 (16)	0.0296 (14)	0.0042 (13)	0.0078 (12)	0.0040 (12)
C24	0.0408 (18)	0.0419 (18)	0.0413 (17)	0.0073 (15)	0.0069 (14)	0.0127 (14)
C25	0.0342 (16)	0.0413 (17)	0.0353 (15)	0.0083 (13)	0.0048 (12)	0.0078 (13)
C26	0.0354 (18)	0.060 (2)	0.050 (2)	0.0091 (16)	−0.0050 (15)	0.0159 (17)
C27	0.0426 (19)	0.0380 (17)	0.0414 (17)	−0.0034 (14)	0.0114 (14)	0.0062 (14)
C28A	0.043 (3)	0.067 (4)	0.060 (4)	−0.008 (3)	−0.003 (3)	0.029 (3)
C29A	0.059 (4)	0.041 (3)	0.080 (5)	−0.002 (3)	0.011 (3)	0.006 (3)
C30A	0.047 (4)	0.081 (5)	0.052 (4)	−0.004 (3)	0.017 (3)	0.010 (3)
C28B	0.064 (8)	0.052 (7)	0.053 (7)	−0.017 (6)	0.015 (6)	−0.011 (5)
C29B	0.058 (8)	0.069 (8)	0.076 (9)	−0.005 (7)	0.005 (6)	0.044 (7)
C30B	0.036 (6)	0.061 (7)	0.077 (8)	0.000 (5)	0.026 (5)	0.013 (6)
C33	0.0346 (16)	0.0337 (15)	0.0324 (15)	0.0019 (13)	0.0130 (12)	0.0025 (12)
C34	0.0338 (17)	0.0471 (19)	0.0416 (17)	0.0067 (14)	0.0182 (13)	0.0070 (14)
C35	0.0268 (15)	0.0380 (16)	0.0433 (17)	0.0059 (13)	0.0123 (12)	0.0051 (13)
C36	0.0289 (17)	0.074 (3)	0.056 (2)	0.0133 (17)	0.0111 (15)	0.0164 (19)
C37	0.0399 (18)	0.0473 (19)	0.0309 (15)	0.0016 (15)	0.0126 (13)	0.0071 (13)
C38	0.0418 (19)	0.0481 (19)	0.0341 (16)	−0.0036 (15)	0.0053 (13)	0.0051 (14)
C39	0.056 (2)	0.056 (2)	0.055 (2)	−0.0096 (19)	0.0012 (18)	0.0247 (19)
C40	0.068 (3)	0.102 (4)	0.0349 (19)	0.015 (3)	0.0226 (19)	0.003 (2)
C43	0.0302 (15)	0.0312 (15)	0.0342 (15)	0.0013 (12)	0.0105 (12)	−0.0025 (12)
C44	0.0397 (18)	0.0398 (17)	0.0275 (14)	0.0008 (14)	0.0129 (12)	−0.0020 (12)
C45	0.0352 (16)	0.0345 (15)	0.0263 (14)	−0.0024 (13)	0.0061 (11)	0.0003 (11)
C46	0.0418 (19)	0.058 (2)	0.0295 (15)	0.0031 (16)	0.0039 (13)	0.0105 (14)
C47	0.0334 (16)	0.0365 (16)	0.0417 (17)	0.0095 (13)	0.0125 (13)	−0.0011 (13)
C48	0.053 (2)	0.044 (2)	0.065 (2)	0.0124 (18)	0.0056 (19)	0.0027 (18)
C49	0.052 (2)	0.077 (3)	0.066 (3)	0.028 (2)	0.031 (2)	0.018 (2)
C50	0.039 (2)	0.057 (2)	0.062 (2)	0.0197 (17)	−0.0040 (17)	−0.0149 (18)
C53	0.0311 (15)	0.0350 (15)	0.0299 (14)	0.0064 (12)	0.0079 (11)	0.0033 (11)
C54	0.0319 (16)	0.0436 (17)	0.0360 (16)	0.0089 (14)	0.0037 (12)	0.0079 (13)
C55	0.0281 (15)	0.0418 (17)	0.0318 (14)	0.0040 (13)	0.0030 (11)	0.0043 (12)
C56	0.0313 (18)	0.052 (2)	0.059 (2)	0.0008 (15)	−0.0073 (15)	0.0017 (17)
C57	0.0404 (18)	0.0342 (16)	0.0398 (17)	0.0049 (14)	0.0038 (13)	0.0025 (13)
C58	0.043 (2)	0.0371 (17)	0.050 (2)	−0.0020 (15)	0.0014 (15)	0.0002 (15)
C59	0.056 (2)	0.045 (2)	0.057 (2)	−0.0010 (18)	0.0055 (18)	0.0184 (17)
C60	0.060 (3)	0.045 (2)	0.062 (2)	0.0214 (19)	0.0019 (19)	−0.0054 (18)
C63	0.0286 (15)	0.0330 (15)	0.0343 (15)	0.0008 (12)	0.0048 (11)	0.0017 (12)
C64	0.0319 (16)	0.0410 (17)	0.0400 (17)	−0.0028 (13)	0.0120 (13)	0.0015 (13)
C65	0.0297 (15)	0.0424 (17)	0.0350 (15)	0.0005 (13)	0.0107 (12)	0.0009 (13)
C66	0.046 (2)	0.055 (2)	0.047 (2)	−0.0077 (17)	0.0220 (16)	−0.0111 (16)
C67	0.0352 (17)	0.0351 (16)	0.0370 (16)	0.0002 (13)	0.0022 (13)	−0.0022 (13)
C68	0.102 (4)	0.042 (2)	0.068 (3)	0.007 (2)	0.004 (3)	0.001 (2)
C69	0.073 (3)	0.072 (3)	0.085 (4)	0.023 (3)	−0.037 (3)	−0.028 (3)
C70	0.097 (4)	0.087 (4)	0.079 (3)	−0.042 (3)	0.050 (3)	−0.043 (3)
B1	0.0228 (15)	0.0347 (17)	0.0286 (15)	0.0042 (13)	0.0009 (12)	−0.0007 (12)
B2	0.0230 (15)	0.0314 (16)	0.0275 (15)	−0.0013 (12)	0.0051 (12)	0.0018 (12)
C1S	0.065 (3)	0.060 (3)	0.061 (3)	−0.002 (2)	0.021 (2)	0.002 (2)
C2S	0.078 (3)	0.052 (2)	0.056 (2)	0.007 (2)	0.023 (2)	0.0016 (19)
C3S	0.093 (4)	0.062 (3)	0.053 (3)	0.006 (3)	0.004 (2)	0.003 (2)
C4S	0.082 (4)	0.079 (4)	0.073 (3)	0.015 (3)	−0.006 (3)	0.018 (3)
C5S	0.096 (4)	0.062 (3)	0.085 (4)	0.024 (3)	0.030 (3)	0.016 (3)
C6S	0.083 (3)	0.052 (2)	0.054 (2)	−0.008 (2)	0.023 (2)	−0.0041 (19)
C7S	0.070 (4)	0.127 (6)	0.163 (8)	−0.017 (4)	0.024 (4)	−0.034 (5)

### Geometric parameters (Å, °)

**Table:**

Yb—N32	2.477 (3)	C36—H36C	0.9800
Yb—N12	2.524 (3)	C37—C38	1.519 (5)
Yb—N22	2.547 (2)	C37—C39	1.537 (5)
Yb—N42	2.555 (2)	C37—C40	1.544 (5)
Yb—N52	2.632 (3)	C38—H38A	0.9800
Yb—N41	2.990 (2)	C38—H38B	0.9800
Yb—H2B	2.29 (4)	C38—H38C	0.9800
N11—C15	1.362 (4)	C39—H39A	0.9800
N11—N12	1.380 (4)	C39—H39B	0.9800
N11—B1	1.554 (4)	C39—H39C	0.9800
N12—C13	1.332 (4)	C40—H40A	0.9800
N21—C25	1.354 (4)	C40—H40B	0.9800
N21—N22	1.387 (3)	C40—H40C	0.9800
N21—B1	1.558 (4)	C43—C44	1.408 (4)
N22—C23	1.341 (4)	C43—C47	1.520 (5)
N31—C35	1.361 (4)	C44—C45	1.372 (5)
N31—N32	1.382 (4)	C44—H44	0.9500
N31—B1	1.560 (4)	C45—C46	1.500 (5)
N32—C33	1.356 (4)	C46—H46A	0.9800
N41—C45	1.363 (4)	C46—H46B	0.9800
N41—N42	1.390 (3)	C46—H46C	0.9800
N41—B2	1.546 (4)	C47—C50	1.518 (5)
N42—C43	1.342 (4)	C47—C49	1.526 (5)
N51—C55	1.355 (4)	C47—C48	1.540 (5)
N51—N52	1.385 (3)	C48—H48A	0.9800
N51—B2	1.553 (4)	C48—H48B	0.9800
N52—C53	1.338 (4)	C48—H48C	0.9800
N61—C65	1.368 (4)	C49—H49A	0.9800
N61—N62	1.369 (3)	C49—H49B	0.9800
N61—B2	1.527 (4)	C49—H49C	0.9800
N62—C63	1.321 (4)	C50—H50A	0.9800
C13—C14	1.396 (5)	C50—H50B	0.9800
C13—C17	1.519 (5)	C50—H50C	0.9800
C14—C15	1.377 (5)	C53—C54	1.401 (4)
C14—H14	0.9500	C53—C57	1.523 (4)
C15—C16	1.490 (5)	C54—C55	1.378 (5)
C16—H16A	0.9800	C54—H54	0.9500
C16—H16B	0.9800	C55—C56	1.498 (5)
C16—H16C	0.9800	C56—H56A	0.9800
C17—C19	1.527 (5)	C56—H56B	0.9800
C17—C18	1.528 (6)	C56—H56C	0.9800
C17—C20	1.538 (5)	C57—C58	1.522 (5)
C18—H18A	0.9800	C57—C59	1.537 (5)
C18—H18B	0.9800	C57—C60	1.548 (5)
C18—H18C	0.9800	C58—H58A	0.9800
C19—H19A	0.9800	C58—H58B	0.9800
C19—H19B	0.9800	C58—H58C	0.9800
C19—H19C	0.9800	C59—H59A	0.9800
C20—H20A	0.9800	C59—H59B	0.9800
C20—H20B	0.9800	C59—H59C	0.9800
C20—H20C	0.9800	C60—H60A	0.9800
C23—C24	1.397 (5)	C60—H60B	0.9800
C23—C27	1.516 (5)	C60—H60C	0.9800
C24—C25	1.377 (5)	C63—C64	1.415 (4)
C24—H24	0.9500	C63—C67	1.519 (4)
C25—C26	1.501 (5)	C64—C65	1.374 (5)
C26—H26A	0.9800	C64—H64	0.9500
C26—H26B	0.9800	C65—C66	1.497 (5)
C26—H26C	0.9800	C66—H66A	0.9800
C27—C28A	1.444 (7)	C66—H66B	0.9800
C27—C29B	1.454 (11)	C66—H66C	0.9800
C27—C30A	1.536 (7)	C67—C70	1.508 (6)
C27—C28B	1.554 (12)	C67—C69	1.512 (6)
C27—C29A	1.588 (7)	C67—C68	1.525 (6)
C27—C30B	1.661 (13)	C68—H68A	0.9800
C28A—H28A	0.9800	C68—H68B	0.9800
C28A—H28B	0.9800	C68—H68C	0.9800
C28A—H28C	0.9800	C69—H69A	0.9800
C29A—H29A	0.9800	C69—H69B	0.9800
C29A—H29B	0.9800	C69—H69C	0.9800
C29A—H29C	0.9800	C70—H70A	0.9800
C30A—H30A	0.9800	C70—H70B	0.9800
C30A—H30B	0.9800	C70—H70C	0.9800
C30A—H30C	0.9800	B1—H1B	1.0000
C28B—H28D	0.9800	B2—H2B	1.16 (3)
C28B—H28E	0.9800	C1S—C2S	1.380 (6)
C28B—H28F	0.9800	C1S—C6S	1.386 (7)
C29B—H29D	0.9800	C1S—C7S	1.476 (9)
C29B—H29E	0.9800	C2S—C3S	1.356 (7)
C29B—H29F	0.9800	C2S—H2S	0.9500
C30B—H30D	0.9800	C3S—C4S	1.347 (8)
C30B—H30E	0.9800	C3S—H3S	0.9500
C30B—H30F	0.9800	C4S—C5S	1.384 (8)
C33—C34	1.391 (5)	C4S—H4S	0.9500
C33—C37	1.522 (5)	C5S—C6S	1.378 (8)
C34—C35	1.372 (5)	C5S—H5S	0.9500
C34—H34	0.9500	C6S—H6S	0.9500
C35—C36	1.500 (5)	C7S—H7SA	0.9800
C36—H36A	0.9800	C7S—H7SB	0.9800
C36—H36B	0.9800	C7S—H7SC	0.9800
			
N32—Yb—N12	87.15 (8)	C35—C36—H36C	109.5
N32—Yb—N22	93.28 (8)	H36A—C36—H36C	109.5
N12—Yb—N22	68.27 (8)	H36B—C36—H36C	109.5
N32—Yb—N42	97.77 (8)	C38—C37—C33	111.9 (3)
N12—Yb—N42	159.83 (9)	C38—C37—C39	110.0 (3)
N22—Yb—N42	91.85 (8)	C33—C37—C39	108.5 (3)
N32—Yb—N52	107.92 (8)	C38—C37—C40	108.6 (3)
N12—Yb—N52	103.07 (8)	C33—C37—C40	109.1 (3)
N22—Yb—N52	156.96 (8)	C39—C37—C40	108.8 (3)
N42—Yb—N52	94.05 (8)	C37—C38—H38A	109.5
N32—Yb—N41	108.81 (7)	C37—C38—H38B	109.5
N12—Yb—N41	162.84 (8)	H38A—C38—H38B	109.5
N22—Yb—N41	115.54 (7)	C37—C38—H38C	109.5
N42—Yb—N41	27.63 (7)	H38A—C38—H38C	109.5
N52—Yb—N41	66.66 (7)	H38B—C38—H38C	109.5
N32—Yb—H2B	153.8 (9)	C37—C39—H39A	109.5
N12—Yb—H2B	118.2 (9)	C37—C39—H39B	109.5
N22—Yb—H2B	101.8 (9)	H39A—C39—H39B	109.5
N42—Yb—H2B	60.9 (9)	C37—C39—H39C	109.5
N52—Yb—H2B	62.5 (9)	H39A—C39—H39C	109.5
N41—Yb—H2B	45.3 (9)	H39B—C39—H39C	109.5
C15—N11—N12	109.6 (2)	C37—C40—H40A	109.5
C15—N11—B1	125.9 (3)	C37—C40—H40B	109.5
N12—N11—B1	122.6 (2)	H40A—C40—H40B	109.5
C13—N12—N11	106.7 (3)	C37—C40—H40C	109.5
C13—N12—Yb	141.1 (2)	H40A—C40—H40C	109.5
N11—N12—Yb	105.18 (17)	H40B—C40—H40C	109.5
C25—N21—N22	109.8 (3)	N42—C43—C44	109.6 (3)
C25—N21—B1	126.8 (3)	N42—C43—C47	122.5 (3)
N22—N21—B1	122.8 (2)	C44—C43—C47	127.7 (3)
C23—N22—N21	106.0 (2)	C45—C44—C43	106.5 (3)
C23—N22—Yb	143.8 (2)	C45—C44—H44	126.7
N21—N22—Yb	109.61 (17)	C43—C44—H44	126.7
C35—N31—N32	109.6 (2)	N41—C45—C44	107.8 (3)
C35—N31—B1	126.1 (3)	N41—C45—C46	123.1 (3)
N32—N31—B1	124.2 (2)	C44—C45—C46	129.1 (3)
C33—N32—N31	106.1 (2)	C45—C46—H46A	109.5
C33—N32—Yb	139.4 (2)	C45—C46—H46B	109.5
N31—N32—Yb	107.80 (17)	H46A—C46—H46B	109.5
C45—N41—N42	109.6 (2)	C45—C46—H46C	109.5
C45—N41—B2	131.9 (3)	H46A—C46—H46C	109.5
N42—N41—B2	117.4 (2)	H46B—C46—H46C	109.5
C45—N41—Yb	143.00 (19)	C50—C47—C43	112.0 (3)
N42—N41—Yb	58.48 (13)	C50—C47—C49	109.3 (3)
B2—N41—Yb	75.48 (15)	C43—C47—C49	110.3 (3)
C43—N42—N41	106.5 (2)	C50—C47—C48	109.2 (3)
C43—N42—Yb	137.2 (2)	C43—C47—C48	107.7 (3)
N41—N42—Yb	93.89 (16)	C49—C47—C48	108.3 (3)
C55—N51—N52	110.2 (3)	C47—C48—H48A	109.5
C55—N51—B2	135.6 (3)	C47—C48—H48B	109.5
N52—N51—B2	114.2 (2)	H48A—C48—H48B	109.5
C53—N52—N51	106.1 (2)	C47—C48—H48C	109.5
C53—N52—Yb	146.8 (2)	H48A—C48—H48C	109.5
N51—N52—Yb	107.03 (17)	H48B—C48—H48C	109.5
C65—N61—N62	110.6 (2)	C47—C49—H49A	109.5
C65—N61—B2	127.9 (3)	C47—C49—H49B	109.5
N62—N61—B2	121.5 (2)	H49A—C49—H49B	109.5
C63—N62—N61	106.1 (2)	C47—C49—H49C	109.5
N12—C13—C14	110.1 (3)	H49A—C49—H49C	109.5
N12—C13—C17	122.5 (3)	H49B—C49—H49C	109.5
C14—C13—C17	127.0 (3)	C47—C50—H50A	109.5
C15—C14—C13	106.2 (3)	C47—C50—H50B	109.5
C15—C14—H14	126.9	H50A—C50—H50B	109.5
C13—C14—H14	126.9	C47—C50—H50C	109.5
N11—C15—C14	107.4 (3)	H50A—C50—H50C	109.5
N11—C15—C16	124.0 (3)	H50B—C50—H50C	109.5
C14—C15—C16	128.6 (3)	N52—C53—C54	110.0 (3)
C15—C16—H16A	109.5	N52—C53—C57	123.9 (3)
C15—C16—H16B	109.5	C54—C53—C57	126.1 (3)
H16A—C16—H16B	109.5	C55—C54—C53	106.4 (3)
C15—C16—H16C	109.5	C55—C54—H54	126.8
H16A—C16—H16C	109.5	C53—C54—H54	126.8
H16B—C16—H16C	109.5	N51—C55—C54	107.3 (3)
C13—C17—C19	111.7 (3)	N51—C55—C56	124.3 (3)
C13—C17—C18	109.9 (4)	C54—C55—C56	128.4 (3)
C19—C17—C18	110.1 (4)	C55—C56—H56A	109.5
C13—C17—C20	107.6 (3)	C55—C56—H56B	109.5
C19—C17—C20	108.6 (4)	H56A—C56—H56B	109.5
C18—C17—C20	108.9 (3)	C55—C56—H56C	109.5
C17—C18—H18A	109.5	H56A—C56—H56C	109.5
C17—C18—H18B	109.5	H56B—C56—H56C	109.5
H18A—C18—H18B	109.5	C58—C57—C53	112.2 (3)
C17—C18—H18C	109.5	C58—C57—C59	110.2 (3)
H18A—C18—H18C	109.5	C53—C57—C59	108.1 (3)
H18B—C18—H18C	109.5	C58—C57—C60	109.2 (3)
C17—C19—H19A	109.5	C53—C57—C60	108.0 (3)
C17—C19—H19B	109.5	C59—C57—C60	109.0 (3)
H19A—C19—H19B	109.5	C57—C58—H58A	109.5
C17—C19—H19C	109.5	C57—C58—H58B	109.5
H19A—C19—H19C	109.5	H58A—C58—H58B	109.5
H19B—C19—H19C	109.5	C57—C58—H58C	109.5
C17—C20—H20A	109.5	H58A—C58—H58C	109.5
C17—C20—H20B	109.5	H58B—C58—H58C	109.5
H20A—C20—H20B	109.5	C57—C59—H59A	109.5
C17—C20—H20C	109.5	C57—C59—H59B	109.5
H20A—C20—H20C	109.5	H59A—C59—H59B	109.5
H20B—C20—H20C	109.5	C57—C59—H59C	109.5
N22—C23—C24	110.3 (3)	H59A—C59—H59C	109.5
N22—C23—C27	123.0 (3)	H59B—C59—H59C	109.5
C24—C23—C27	126.4 (3)	C57—C60—H60A	109.5
C25—C24—C23	106.0 (3)	C57—C60—H60B	109.5
C25—C24—H24	127.0	H60A—C60—H60B	109.5
C23—C24—H24	127.0	C57—C60—H60C	109.5
N21—C25—C24	107.9 (3)	H60A—C60—H60C	109.5
N21—C25—C26	125.1 (3)	H60B—C60—H60C	109.5
C24—C25—C26	127.0 (3)	N62—C63—C64	111.0 (3)
C25—C26—H26A	109.5	N62—C63—C67	120.6 (3)
C25—C26—H26B	109.5	C64—C63—C67	128.4 (3)
H26A—C26—H26B	109.5	C65—C64—C63	105.1 (3)
C25—C26—H26C	109.5	C65—C64—H64	127.4
H26A—C26—H26C	109.5	C63—C64—H64	127.4
H26B—C26—H26C	109.5	N61—C65—C64	107.2 (3)
C28A—C27—C29B	131.5 (7)	N61—C65—C66	122.2 (3)
C28A—C27—C23	113.6 (4)	C64—C65—C66	130.5 (3)
C29B—C27—C23	114.8 (6)	C65—C66—H66A	109.5
C28A—C27—C30A	112.1 (5)	C65—C66—H66B	109.5
C29B—C27—C30A	49.8 (7)	H66A—C66—H66B	109.5
C23—C27—C30A	107.2 (4)	C65—C66—H66C	109.5
C28A—C27—C28B	48.7 (6)	H66A—C66—H66C	109.5
C29B—C27—C28B	112.3 (9)	H66B—C66—H66C	109.5
C23—C27—C28B	111.9 (5)	C70—C67—C69	110.9 (4)
C30A—C27—C28B	140.8 (6)	C70—C67—C63	111.4 (3)
C28A—C27—C29A	110.0 (5)	C69—C67—C63	109.9 (3)
C29B—C27—C29A	56.3 (7)	C70—C67—C68	108.1 (4)
C23—C27—C29A	107.9 (4)	C69—C67—C68	107.1 (4)
C30A—C27—C29A	105.7 (5)	C63—C67—C68	109.3 (3)
C28B—C27—C29A	64.5 (6)	C67—C68—H68A	109.5
C28A—C27—C30B	54.1 (6)	C67—C68—H68B	109.5
C29B—C27—C30B	108.3 (8)	H68A—C68—H68B	109.5
C23—C27—C30B	106.7 (5)	C67—C68—H68C	109.5
C30A—C27—C30B	63.7 (6)	H68A—C68—H68C	109.5
C28B—C27—C30B	101.9 (8)	H68B—C68—H68C	109.5
C29A—C27—C30B	145.4 (6)	C67—C69—H69A	109.5
C27—C28A—H28A	109.5	C67—C69—H69B	109.5
C27—C28A—H28B	109.5	H69A—C69—H69B	109.5
H28A—C28A—H28B	109.5	C67—C69—H69C	109.5
C27—C28A—H28C	109.5	H69A—C69—H69C	109.5
H28A—C28A—H28C	109.5	H69B—C69—H69C	109.5
H28B—C28A—H28C	109.5	C67—C70—H70A	109.5
C27—C29A—H29A	109.5	C67—C70—H70B	109.5
C27—C29A—H29B	109.5	H70A—C70—H70B	109.5
H29A—C29A—H29B	109.5	C67—C70—H70C	109.5
C27—C29A—H29C	109.5	H70A—C70—H70C	109.5
H29A—C29A—H29C	109.5	H70B—C70—H70C	109.5
H29B—C29A—H29C	109.5	N11—B1—N21	110.2 (2)
C27—C30A—H30A	109.5	N11—B1—N31	111.8 (2)
C27—C30A—H30B	109.5	N21—B1—N31	113.0 (2)
H30A—C30A—H30B	109.5	N11—B1—H1B	107.2
C27—C30A—H30C	109.5	N21—B1—H1B	107.2
H30A—C30A—H30C	109.5	N31—B1—H1B	107.2
H30B—C30A—H30C	109.5	N61—B2—N41	112.3 (2)
C27—C28B—H28D	109.5	N61—B2—N51	113.5 (2)
C27—C28B—H28E	109.5	N41—B2—N51	110.9 (2)
H28D—C28B—H28E	109.5	N61—B2—H2B	110.7 (18)
C27—C28B—H28F	109.5	N41—B2—H2B	103.3 (18)
H28D—C28B—H28F	109.5	N51—B2—H2B	105.5 (17)
H28E—C28B—H28F	109.5	C2S—C1S—C6S	117.7 (5)
C27—C29B—H29D	109.5	C2S—C1S—C7S	121.1 (5)
C27—C29B—H29E	109.5	C6S—C1S—C7S	121.2 (5)
H29D—C29B—H29E	109.5	C3S—C2S—C1S	121.0 (5)
C27—C29B—H29F	109.5	C3S—C2S—H2S	119.5
H29D—C29B—H29F	109.5	C1S—C2S—H2S	119.5
H29E—C29B—H29F	109.5	C4S—C3S—C2S	121.7 (5)
C27—C30B—H30D	109.5	C4S—C3S—H3S	119.2
C27—C30B—H30E	109.5	C2S—C3S—H3S	119.2
H30D—C30B—H30E	109.5	C3S—C4S—C5S	119.0 (5)
C27—C30B—H30F	109.5	C3S—C4S—H4S	120.5
H30D—C30B—H30F	109.5	C5S—C4S—H4S	120.5
H30E—C30B—H30F	109.5	C6S—C5S—C4S	119.8 (5)
N32—C33—C34	109.8 (3)	C6S—C5S—H5S	120.1
N32—C33—C37	123.3 (3)	C4S—C5S—H5S	120.1
C34—C33—C37	126.7 (3)	C5S—C6S—C1S	120.7 (5)
C35—C34—C33	106.5 (3)	C5S—C6S—H6S	119.7
C35—C34—H34	126.7	C1S—C6S—H6S	119.7
C33—C34—H34	126.7	C1S—C7S—H7SA	109.5
N31—C35—C34	108.0 (3)	C1S—C7S—H7SB	109.5
N31—C35—C36	123.8 (3)	H7SA—C7S—H7SB	109.5
C34—C35—C36	128.3 (3)	C1S—C7S—H7SC	109.5
C35—C36—H36A	109.5	H7SA—C7S—H7SC	109.5
C35—C36—H36B	109.5	H7SB—C7S—H7SC	109.5
H36A—C36—H36B	109.5		
			
C15—N11—N12—C13	−1.4 (3)	C24—C23—C27—C28A	−172.3 (5)
B1—N11—N12—C13	163.8 (3)	N22—C23—C27—C29B	−163.5 (8)
C15—N11—N12—Yb	155.98 (19)	C24—C23—C27—C29B	10.3 (9)
B1—N11—N12—Yb	−38.8 (3)	N22—C23—C27—C30A	−110.5 (5)
N32—Yb—N12—C13	124.1 (4)	C24—C23—C27—C30A	63.3 (5)
N22—Yb—N12—C13	−141.2 (4)	N22—C23—C27—C28B	67.0 (7)
N42—Yb—N12—C13	−131.0 (4)	C24—C23—C27—C28B	−119.2 (7)
N52—Yb—N12—C13	16.4 (4)	N22—C23—C27—C29A	136.1 (4)
N41—Yb—N12—C13	−34.8 (6)	C24—C23—C27—C29A	−50.1 (5)
N32—Yb—N12—N11	−19.97 (19)	N22—C23—C27—C30B	−43.6 (6)
N22—Yb—N12—N11	74.70 (18)	C24—C23—C27—C30B	130.2 (6)
N42—Yb—N12—N11	84.9 (3)	N31—N32—C33—C34	0.9 (3)
N52—Yb—N12—N11	−127.71 (18)	Yb—N32—C33—C34	−144.7 (3)
N41—Yb—N12—N11	−178.9 (2)	N31—N32—C33—C37	−174.2 (3)
C25—N21—N22—C23	0.7 (3)	Yb—N32—C33—C37	40.2 (5)
B1—N21—N22—C23	−171.0 (3)	N32—C33—C34—C35	−0.7 (4)
C25—N21—N22—Yb	−172.4 (2)	C37—C33—C34—C35	174.2 (3)
B1—N21—N22—Yb	15.9 (3)	N32—N31—C35—C34	0.5 (4)
N32—Yb—N22—C23	−146.8 (3)	B1—N31—C35—C34	−174.7 (3)
N12—Yb—N22—C23	127.6 (4)	N32—N31—C35—C36	−179.3 (3)
N42—Yb—N22—C23	−48.9 (3)	B1—N31—C35—C36	5.5 (5)
N52—Yb—N22—C23	55.9 (4)	C33—C34—C35—N31	0.1 (4)
N41—Yb—N22—C23	−34.2 (4)	C33—C34—C35—C36	179.8 (4)
N32—Yb—N22—N21	21.95 (18)	N32—C33—C37—C38	−25.9 (4)
N12—Yb—N22—N21	−63.67 (17)	C34—C33—C37—C38	159.9 (3)
N42—Yb—N22—N21	119.84 (17)	N32—C33—C37—C39	95.5 (4)
N52—Yb—N22—N21	−135.3 (2)	C34—C33—C37—C39	−78.7 (4)
N41—Yb—N22—N21	134.62 (16)	N32—C33—C37—C40	−146.1 (3)
C35—N31—N32—C33	−0.9 (3)	C34—C33—C37—C40	39.7 (5)
B1—N31—N32—C33	174.5 (3)	N41—N42—C43—C44	0.5 (3)
C35—N31—N32—Yb	156.5 (2)	Yb—N42—C43—C44	−114.5 (3)
B1—N31—N32—Yb	−28.2 (3)	N41—N42—C43—C47	−174.5 (3)
N12—Yb—N32—C33	−162.7 (3)	Yb—N42—C43—C47	70.4 (4)
N22—Yb—N32—C33	129.3 (3)	N42—C43—C44—C45	0.2 (4)
N42—Yb—N32—C33	36.9 (3)	C47—C43—C44—C45	174.9 (3)
N52—Yb—N32—C33	−59.9 (3)	N42—N41—C45—C44	1.2 (3)
N41—Yb—N32—C33	10.9 (3)	B2—N41—C45—C44	−166.4 (3)
N12—Yb—N32—N31	52.01 (18)	Yb—N41—C45—C44	64.5 (4)
N22—Yb—N32—N31	−16.02 (18)	N42—N41—C45—C46	−175.9 (3)
N42—Yb—N32—N31	−108.34 (17)	B2—N41—C45—C46	16.6 (5)
N52—Yb—N32—N31	154.82 (16)	Yb—N41—C45—C46	−112.5 (4)
N41—Yb—N32—N31	−134.43 (16)	C43—C44—C45—N41	−0.8 (4)
N32—Yb—N41—C45	−10.9 (4)	C43—C44—C45—C46	176.0 (3)
N12—Yb—N41—C45	146.8 (3)	N42—C43—C47—C50	−30.1 (5)
N22—Yb—N41—C45	−114.2 (3)	C44—C43—C47—C50	155.9 (3)
N42—Yb—N41—C45	−80.8 (4)	N42—C43—C47—C49	−152.0 (3)
N52—Yb—N41—C45	91.1 (3)	C44—C43—C47—C49	34.0 (5)
N32—Yb—N41—N42	69.95 (17)	N42—C43—C47—C48	90.0 (4)
N12—Yb—N41—N42	−132.3 (3)	C44—C43—C47—C48	−84.1 (4)
N22—Yb—N41—N42	−33.33 (17)	N51—N52—C53—C54	0.7 (3)
N52—Yb—N41—N42	171.90 (17)	Yb—N52—C53—C54	179.5 (3)
N32—Yb—N41—B2	−154.31 (16)	N51—N52—C53—C57	−179.4 (3)
N12—Yb—N41—B2	3.4 (3)	Yb—N52—C53—C57	−0.7 (5)
N22—Yb—N41—B2	102.41 (17)	N52—C53—C54—C55	−0.5 (4)
N42—Yb—N41—B2	135.7 (2)	C57—C53—C54—C55	179.6 (3)
N52—Yb—N41—B2	−52.36 (16)	N52—N51—C55—C54	0.3 (3)
C45—N41—N42—C43	−1.0 (3)	B2—N51—C55—C54	−177.3 (3)
B2—N41—N42—C43	168.5 (3)	N52—N51—C55—C56	−178.7 (3)
Yb—N41—N42—C43	−141.9 (2)	B2—N51—C55—C56	3.7 (6)
C45—N41—N42—Yb	140.9 (2)	C53—C54—C55—N51	0.1 (3)
B2—N41—N42—Yb	−49.5 (2)	C53—C54—C55—C56	179.1 (3)
N32—Yb—N42—C43	3.3 (3)	N52—C53—C57—C58	−4.8 (4)
N12—Yb—N42—C43	−99.8 (4)	C54—C53—C57—C58	175.1 (3)
N22—Yb—N42—C43	−90.3 (3)	N52—C53—C57—C59	117.0 (3)
N52—Yb—N42—C43	112.0 (3)	C54—C53—C57—C59	−63.2 (4)
N41—Yb—N42—C43	119.4 (4)	N52—C53—C57—C60	−125.2 (3)
N32—Yb—N42—N41	−116.17 (16)	C54—C53—C57—C60	54.6 (4)
N12—Yb—N42—N41	140.8 (2)	N61—N62—C63—C64	−0.1 (4)
N22—Yb—N42—N41	150.26 (16)	N61—N62—C63—C67	179.5 (3)
N52—Yb—N42—N41	−7.45 (16)	N62—C63—C64—C65	0.3 (4)
C55—N51—N52—C53	−0.6 (3)	C67—C63—C64—C65	−179.3 (3)
B2—N51—N52—C53	177.5 (2)	N62—N61—C65—C64	0.3 (4)
C55—N51—N52—Yb	−179.92 (19)	B2—N61—C65—C64	179.5 (3)
B2—N51—N52—Yb	−1.8 (3)	N62—N61—C65—C66	−179.0 (3)
N32—Yb—N52—C53	−44.1 (4)	B2—N61—C65—C66	0.2 (5)
N12—Yb—N52—C53	47.1 (4)	C63—C64—C65—N61	−0.4 (4)
N22—Yb—N52—C53	112.0 (4)	C63—C64—C65—C66	178.9 (4)
N42—Yb—N52—C53	−143.6 (4)	N62—C63—C67—C70	0.8 (5)
N41—Yb—N52—C53	−147.4 (4)	C64—C63—C67—C70	−179.8 (5)
N32—Yb—N52—N51	134.66 (17)	N62—C63—C67—C69	−122.6 (4)
N12—Yb—N52—N51	−134.12 (17)	C64—C63—C67—C69	56.9 (5)
N22—Yb—N52—N51	−69.3 (3)	N62—C63—C67—C68	120.1 (4)
N42—Yb—N52—N51	35.14 (17)	C64—C63—C67—C68	−60.4 (5)
N41—Yb—N52—N51	31.38 (16)	C15—N11—B1—N21	125.8 (3)
C65—N61—N62—C63	−0.1 (3)	N12—N11—B1—N21	−36.9 (4)
B2—N61—N62—C63	−179.4 (3)	C15—N11—B1—N31	−107.6 (3)
N11—N12—C13—C14	1.2 (4)	N12—N11—B1—N31	89.7 (3)
Yb—N12—C13—C14	−142.6 (3)	C25—N21—B1—N11	−118.6 (3)
N11—N12—C13—C17	−172.2 (3)	N22—N21—B1—N11	51.7 (3)
Yb—N12—C13—C17	44.0 (5)	C25—N21—B1—N31	115.6 (3)
N12—C13—C14—C15	−0.6 (4)	N22—N21—B1—N31	−74.2 (3)
C17—C13—C14—C15	172.5 (3)	C35—N31—B1—N11	134.9 (3)
N12—N11—C15—C14	1.1 (3)	N32—N31—B1—N11	−39.6 (4)
B1—N11—C15—C14	−163.5 (3)	C35—N31—B1—N21	−100.1 (3)
N12—N11—C15—C16	−177.4 (3)	N32—N31—B1—N21	85.3 (3)
B1—N11—C15—C16	18.0 (5)	C65—N61—B2—N41	162.1 (3)
C13—C14—C15—N11	−0.3 (4)	N62—N61—B2—N41	−18.8 (4)
C13—C14—C15—C16	178.0 (3)	C65—N61—B2—N51	−71.2 (4)
N12—C13—C17—C19	−20.8 (5)	N62—N61—B2—N51	108.0 (3)
C14—C13—C17—C19	167.0 (4)	C45—N41—B2—N61	57.9 (4)
N12—C13—C17—C18	−143.3 (3)	N42—N41—B2—N61	−108.9 (3)
C14—C13—C17—C18	44.4 (5)	Yb—N41—B2—N61	−151.0 (2)
N12—C13—C17—C20	98.2 (4)	C45—N41—B2—N51	−70.3 (4)
C14—C13—C17—C20	−74.0 (5)	N42—N41—B2—N51	123.0 (3)
N21—N22—C23—C24	−0.9 (3)	Yb—N41—B2—N51	80.9 (2)
Yb—N22—C23—C24	168.1 (2)	C55—N51—B2—N61	−20.9 (5)
N21—N22—C23—C27	173.8 (3)	N52—N51—B2—N61	161.6 (2)
Yb—N22—C23—C27	−17.2 (5)	C55—N51—B2—N41	106.6 (4)
N22—C23—C24—C25	0.8 (4)	N52—N51—B2—N41	−70.9 (3)
C27—C23—C24—C25	−173.7 (3)	C6S—C1S—C2S—C3S	−1.6 (7)
N22—N21—C25—C24	−0.2 (4)	C7S—C1S—C2S—C3S	179.3 (6)
B1—N21—C25—C24	171.1 (3)	C1S—C2S—C3S—C4S	2.1 (8)
N22—N21—C25—C26	−179.9 (3)	C2S—C3S—C4S—C5S	−0.3 (8)
B1—N21—C25—C26	−8.5 (5)	C3S—C4S—C5S—C6S	−1.9 (8)
C23—C24—C25—N21	−0.3 (4)	C4S—C5S—C6S—C1S	2.4 (8)
C23—C24—C25—C26	179.3 (3)	C2S—C1S—C6S—C5S	−0.6 (7)
N22—C23—C27—C28A	13.9 (6)	C7S—C1S—C6S—C5S	178.5 (6)

### Hydrogen-bond geometry (Å, °)

**Table:**

*D*—H···*A*	*D*—H	H···*A*	*D*···*A*	*D*—H···*A*
B2—H2B···Yb	1.16 (3)	2.29 (4)	3.002 (3)	118 (2)
